# Introducing physician associates to hospital patients: Development and feasibility testing of a patient experience‐based intervention

**DOI:** 10.1111/hex.13149

**Published:** 2020-11-25

**Authors:** Francesca Taylor, Jonathan Ogidi, Rakhee Chauhan, Zeena Ladva, Sally Brearley, Vari M. Drennan

**Affiliations:** ^1^ Joint Faculty of Kingston University and St George’s University of London, St George's University of London London UK; ^2^ St George’s University Hospitals NHS Foundation Trust London UK

**Keywords:** co‐design, hospital patient information, patient and public involvement, patient experience, physician assistants, physician associates, qualitative research

## Abstract

**Background:**

Physician associates (PAs) are one of many new mid‐level health practitioner roles being introduced worldwide. They are a recent innovation in English hospitals. Patient confusion with novel mid‐level practitioner titles and roles is well documented, alongside evidence of a positive association between patients’ ability to identify practitioners and patient satisfaction. No prior research developed an intervention to introduce PAs or any other new practitioner role to hospital patients.

**Objective:**

To develop, with patient and public involvement and engagement (PPIE), an intervention for introducing the PA role to hospital patients, and to test feasibility.

**Methods:**

Intervention development was underpinned by an experience‐based co‐design approach. Workshop participants generated ideas for introducing PAs, subsequently explored in semi‐structured interviews with hospital patients (n = 13). Interview findings were used by participants in a second workshop to design the intervention. Feasibility of the intervention was assessed in relation to its acceptability and efficacy using semi‐structured interviews with hospital patients (n = 20) and PAs (n = 3).

**Results:**

The intervention developed was a patient information leaflet. It was considered feasible to use in the hospital setting, helpful to patients in understanding the PA role and acceptable to both patients and PAs. The intervention was also appreciated by patients for providing reassurance of care and support.

**Conclusions:**

An experience‐based co‐design approach enabled development of an intervention tailored to patients’ experiential preferences. Positive evidence of feasibility and utility is encouraging, supporting future larger‐scale testing.

**Patient and public contribution:**

PPIE representatives were involved in the study design, intervention development and data interpretation.

## 1 INTRODUCTION

Health‐care systems worldwide are developing new health professional roles delivered by mid‐level practitioners^1^, ^2^ to help address the quadruple aim of improving population health, patient experience and staff work environments, and containing costs.^3^ While some new roles such as nurse practitioners incorporate existing health‐care professionals, others are relatively novel professional groups such as physician assistants, known as physician associates (PAs) in the United Kingdom. PAs have a fifty‐year history in the United States (US) but are a recent introduction in many more countries globally.^4^, ^5^ In England, PAs have been introduced into the National Health Service (NHS) as postgraduate, medically trained professionals undertaking medical histories, physical examinations, diagnosis and treatment within their scope of practice, under doctor supervision in medical/surgical teams.^4^, ^6^ Although small numbers of PAs currently work in NHS acute hospitals in England, the numbers will increase substantially from 2020 onwards as a result of government funding for PA education.^7^

Patient confusion with new mid‐level practitioner titles and roles has been well documented,^8^, ^9^, ^10^, ^11^ alongside evidence that the novel terminology can be difficult to interpret and is sometimes misleading.^8^, ^10^ Existing studies in England report poor recognition and comprehension of the PA role among hospital patients.^12^, ^13^ They are often confused by the title; its meaning is not immediately obvious and needs explanation. Furthermore, patients can mistakenly perceive PAs to be doctors and express concerns when made aware of the misconception. To prevent confusion, explanatory patient information about the role was considered necessary and beneficial.^13^ A study in the Netherlands, where the role is also unfamiliar, similarly identified that hospital staff thought patients were unaware whether they had seen a PA or doctor.^14^ Moreover, a US study undertaken over 30 years after the introduction of PAs found that emergency department inpatients needed better information about the role; patient confusion as to provider identity was reportedly associated with often covert physician substitution.^15^ There is also evidence of the potential for patients’ trust and confidence in the PA to be affected by lack of transparency,^13^ with possible adverse implications for the PA‐patient relationship.^16^

Some studies have shown a positive association between hospital patients’ ability to identify clinicians involved in their care, and patient‐clinician communication and patient experience and satisfaction.^17^, ^18^, ^19^ Explanatory theories suggest reduced psychological stress experienced by the hospitalized patients.^20^, ^21^ Evidence is inconclusive that interventions such as facecards, and use of whiteboards can improve clinician identification and understanding of roles among inpatients.^22^, ^23^ However, these interventions were conceived by hospital staff or researchers and not with patients. Patient and public involvement and engagement (PPIE) in health‐care service improvement studies has been shown to develop interventions that are more appropriate and responsive to patients’ needs,^24^, ^25^, ^26^ and positively influence quality outcome data.^27^, ^28^ No studies, to our knowledge, have examined interventions for introducing PAs or any other new professional role to hospital patients. To address these evidence gaps, the aim of this study was to develop, with PPIE, an intervention for introducing the PA role to hospital patients and to test feasibility. The research questions addressed were as follows: what is the preferred method of introducing the PA role? Is the preferred method feasible to use, helpful or otherwise in understanding the PA role and acceptable to hospital patients and PAs?

## 2 METHODS

### 2.1 Study design

**Figure 1:**
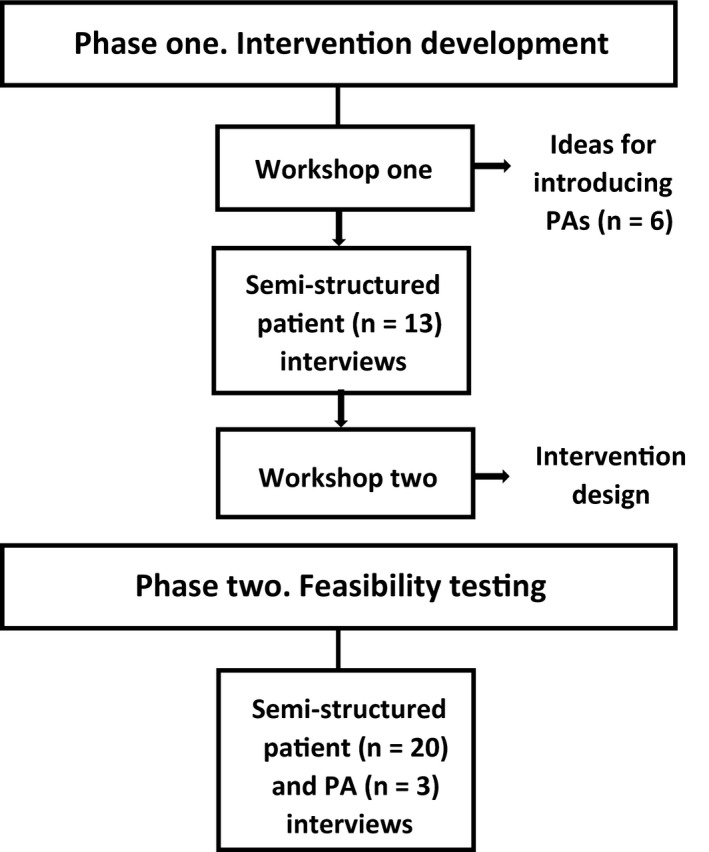
Process of development and feasibility testing of the intervention

The study was undertaken in two phases: (1) intervention development and (2) feasibility testing (Figure 1), informed by interpretive methodology.^29^, ^30^ The strength of the methodology was that it allowed for focus on exploration and understanding of participants’ preferences for introducing the PA role to hospital patients (phase one), and consideration of context in understanding the experiences of patients and PAs in feasibility testing of the intervention (phase two). Experiences being essentially linked to context, in terms of time, location, and the mindset of the participant.^31^ The study took place between November 2018 and May 2019 in one English NHS acute hospital. The study site was an urban teaching hospital with 1300 inpatient beds.

The aim was to include patient and public involvement and engagement (PPIE) representatives as equal partners with researchers and PAs in the study. A recent review of frameworks for supporting PPIE^32^ identified several that were partnership‐focused, as distinct from those that were more power‐focused, report‐focused or concerned with priority setting. Partnership‐focused frameworks, for example, the INVOLVE values and principles framework,^33^ commonly emphasize governance structures, inclusive opportunities, transparency, communication showing that researchers have responded to comments, and support and training. We used this guidance in setting up the study.

An established Patient Research Expert Group of PPIE representatives, facilitated by SB, in the University's Centre for Public Engagement, was closely involved in the study design. All the PPIE representatives had recent lived experience of being a hospital inpatient or were carers for people who had recently been hospital inpatients. They also had prior experience of involvement in health‐related co‐design projects. The PPIE representatives (n = 15) were sent an email invitation by the study researchers to participate throughout the study, with information on the study aims and what participation involved. Eight PPIE representatives volunteered and consented to participate.

PAs were eligible if currently employed in the study site. Eligible PAs (n = 23) were invited by the lead PA to participate in both study phases. Contact details of PAs expressing interest in participation were passed to study researchers who sent the PAs a participant information sheet and consent form. Four PAs consented to participate.

#### 2.1.1 Phase one: Intervention development

Development of the intervention was underpinned by an adapted experience‐based co‐design approach.^34^ It was undertaken in three stages: workshop one, semi‐structured patient interviews and workshop two.

##### Workshop one

Workshop^35^ one with the volunteer PPIE representatives was facilitated by a study researcher (FT). The goal was to generate six ideas for introducing PAs to hospital patients. After discussing the PPIE representatives’ initial views, the researcher introduced 14 different outline ideas for introducing PAs. The ideas had been developed by the study researchers from previous approaches used to improve clinician identification and understanding of clinical roles, reported in the literature.^23^, ^36^ For example, a poster showing the faces and titles of different hospital medical/surgical team members. The outline ideas were used to stimulate discussion and prompt idea generation. Workshop participants were asked to reach consensus on their choices from the ideas to introduce PAs to hospital patients that they generated. The six ideas chosen were as follows: the PA title on badge and lanyard; a standardized script for PAs to introduce themselves; a poster describing the PA role; a poster with the photograph, name and title of all medical/clinical team members including the PAs; a leaflet describing the PA role; and a leaflet with the photograph of a PA and a description of their role written in the first person. These six ideas were then shared with the PA study participants, who modified some wording to ensure accuracy on PA training.

##### Semi‐structured patient interviews

Hospital patients’ responses to the six ideas for introducing PAs were explored qualitatively to enable insights into their attitudes and preferences. Guidance for ensuring quality when undertaking qualitative research^37^, ^38^ was utilized to assess reliability in methodological approach. Semi‐structured interviews were undertaken face‐to‐face with participants in hospital during episodes of care. Eligibility criteria specified consenting adult inpatients—and adult representatives of inpatients—aged 16 years or over, receiving care from a PA study participant. Patients were excluded if not clinically well enough or lacking capacity to give informed consent. Sampling was purposive to provide diversity by patient age, gender and ethnic group, and medical/surgical speciality of the PA. Patients who met the eligibility criteria were initially identified by a PA study participant and then approached by the researcher. Patients expressing interest were given an information sheet outlining the study purpose and what participation would involve. They could choose an individual personal interview or nominate a friend or family member present in the hospital as a representative to be interviewed on their behalf. To ensure maximum confidentiality, consent was taken by the researcher and interviews undertaken in a room separate from the main inpatient ward or where not possible, at the bedside with curtain partition. A total of 19 patients and patient representatives were introduced to the researcher, and 13 (68%) consented to participate in a single interview. Five patients and one patient representative withdrew before consenting due to patient ill‐health.

The six ideas for introducing PAs to hospital patients were shown individually to each participant in written and/or visual form on A4‐size paper. For example, to show the idea for the PA title on badge and lanyard, a picture of a clinician wearing a badge and lanyard with the words ‘Physician associate’ was used. To avoid response‐order bias, the order in which the ideas were shown to participants was rotated to a structured format. A topic guide was developed based on the study aims and informed by the literature and input from the study PPIE representatives. It included questions about patients’ feelings and attitudes in relation to each idea and their preferences in terms of mode, format, content and timing. Interviews lasted between 18 and 45 minutes (mean 27) and were audio‐recorded and transcribed.

##### Workshop two

A second workshop, facilitated by SB, focused on designing an intervention to introduce PAs. Workshop participants included the volunteer PPIE representatives, the lead PA participant, and a study researcher (FT). Findings from the semi‐structured patient interviews with hospital patients were shared by the study researcher and then discussed and interpreted together with the participants. Consequent to this exploration of the findings there was consensual agreement among participants that the intervention should be a patient information leaflet (PIL). Participants were then facilitated to use the interview findings to construct components of the PIL design. Following the workshop, the researcher circulated a PIL prototype to participants for verification that it reflected their proposed design.

#### 2.1.2 Phase two: Feasibility testing

Feasibility of the intervention, a PIL, was assessed in relation to its acceptability and its efficacy in being helpful or otherwise for hospital patients in understanding the PA role.^39^ We used evidence‐based guidance for evaluating PILs to support data analysis and interpretation.^40^ Semi‐structured interviews were undertaken face‐to‐face with PA and patient participants. PA participants were asked to use the intervention for each inpatient they attended routinely over a three‐week period. Three PAs participated and consented to be interviewed; one PA withdrew from the study after phase one due to work pressures. Eligibility criteria for patients were the same as for the semi‐structured patient interviews in phase one, with the additional specification that patients had received the intervention. The same patient sampling, recruitment and consent procedures were also followed. Patient participant interviews took place one‐three days after they had received the intervention. Twenty‐four patients and patient representatives were introduced to the researcher and 20 (83%) consented to participate in a single interview. Four patients withdrew prior to consenting due to ill‐health.

The interviews consisted of open‐ended questions with supplementary prompts to enable key areas of interest to be explored without being overly prescriptive about content and direction.^41^ Topic guides were based on the study aims and informed by evidence‐based guidance for evaluating PILs^40^ and input from the study PPIE representatives. The patient topic guide included questions on how participants experienced and responded to the intervention and any suggested improvements. The PA topic guide asked how participants introduced the intervention and in which context, and about any perceived benefits/disadvantages. Interviews lasted between 12 and 47 minutes (mean 29) and were audio‐recorded and transcribed.

### 2.2 Data analysis

#### 2.2.1 Phase one: Intervention development

Emergent data from the first workshop were analysed inductively by the researcher (FT). Results were circulated among participants for verification that they accurately reflected workshop discussions.

A framework approach^42^, ^43^ was used to code and categorize interview data. Transcripts were analysed by FT employing open coding and constant comparison.^43^ An initial framework was developed from the codes and categories after scrutiny and discussion with the principal investigator (VMD), who had read a sub‐sample of transcripts. Together the researchers asked questions of the data to assist identification of category properties. Verbatim patient responses were then entered onto a spreadsheet with the coding framework. This process was undertaken independently by FT supplemented by collaborative discussion with VMD to reach consensus and confirm categories.

In the second workshop, data analysis was undertaken in situ by FT together with the participants, drawing together their ideas and opinions for the intervention design.

#### 2.2.2 Phase two: Feasibility testing

The patient and PA interview data sets were initially analysed separately using thematic analysis (FT).^42^ The analysis was informed by the study topic guide and evidence‐based guidance for evaluating PILs.^40^ Data were broken down using line‐by‐line coding and codes clustered manually to identify preliminary categories based on issues and themes. These were scrutinized and discussed with VMD who read transcripts from a sub‐sample of interviews from each data set. Two separate frameworks were developed from the analyses, one for the patient data and one for the PA data, together with code‐books, and used to structure verbatim responses onto spreadsheets. The codes and themes included in each of the frameworks were refined and elaborated collectively with data collection from further interviews. As sequential analysis progressed, significant data were compressed to adhere around key analytic themes. Where data did not fit existing themes, new ones were developed or existing ones modified, until all data were coded by theme. A further stage of synthesis was undertaken (FT, VMD) to describe and interpret findings, looking for triangulation of themes, patterns and plausible explanations across the two data sets, before confirmation of themes.

## 3 RESULTS

### 3.1 Phase one: Intervention development

In the first workshop, most participants described a lack of information about the health‐care providers who delivered their hospital care. However, they perceived being informed about who was attending them to be an integral component of patient‐centred hospital care. Particular concern was expressed about the lack of transparency involved if PAs were perceived to be doctors. They considered it important that patients be informed about and understand the PA role in different hospital settings.

Eleven hospital patients and two patient representatives participated in semi‐structured interviews (Table 1) that explored their responses to the six ideas for introducing PAs developed by workshop participants. The patients interviewed ranged in age from 34 to 81 years. For one participant English was a second language. Participants were generally very supportive of information being provided about the PA role since the title was unfamiliar, generating uncertainty about who a PA was and what they did. Features of information provision particularly favoured were as follows: patient‐friendly, jargon‐free language explaining that PAs are fully trained and what they are qualified to do; reassurance that PAs are part of a medical team; and spoken personal introduction supported by written information.

**Table 1:** Semi‐structured interview participants in each study phase

	Phase one: Intervention development (n = 13)	Phase two: Feasibility testing (n = 20)
*Participant type*		
Patient	11	16
Patient representative	2	4
*Gender*		
Female	9	8
Male	4	12
*Department*		
Acute stroke	3	7
Orthopaedic	8	11
Surgical oncology	2	2

There were variable and individualistic responses from participants to the different ideas to introduce PAs that they were shown, reflective of diverse information needs. Nevertheless, a hand‐size information leaflet was preferred, consistently generating positive views. Participants spoke about the benefits of choice in relation to when information was accessed while an inpatient, recognizing that their capacity and inclination to absorb information could fluctuate. Perceiving a leaflet to offer choice as to when it could be read and referenced was therefore attractive to many participants. Additionally, some participants mentioned that a hand‐held information leaflet was more person‐focused and convenient to read than accessing information from a poster.

I haven't been out of my bed for two weeks so unless it's somewhere in my view it's no good to me…a leaflet you can read as right in front of you. (Participant 9, male, orthopaedic)

Some participants mentioned the perceived need for a leaflet to be made available in translated form for patients whose first language was not English. Others suggested a leaflet had the advantage that if unable to be read by a patient, it could be given to a family member who was able to read the information.

**Figure 2:**
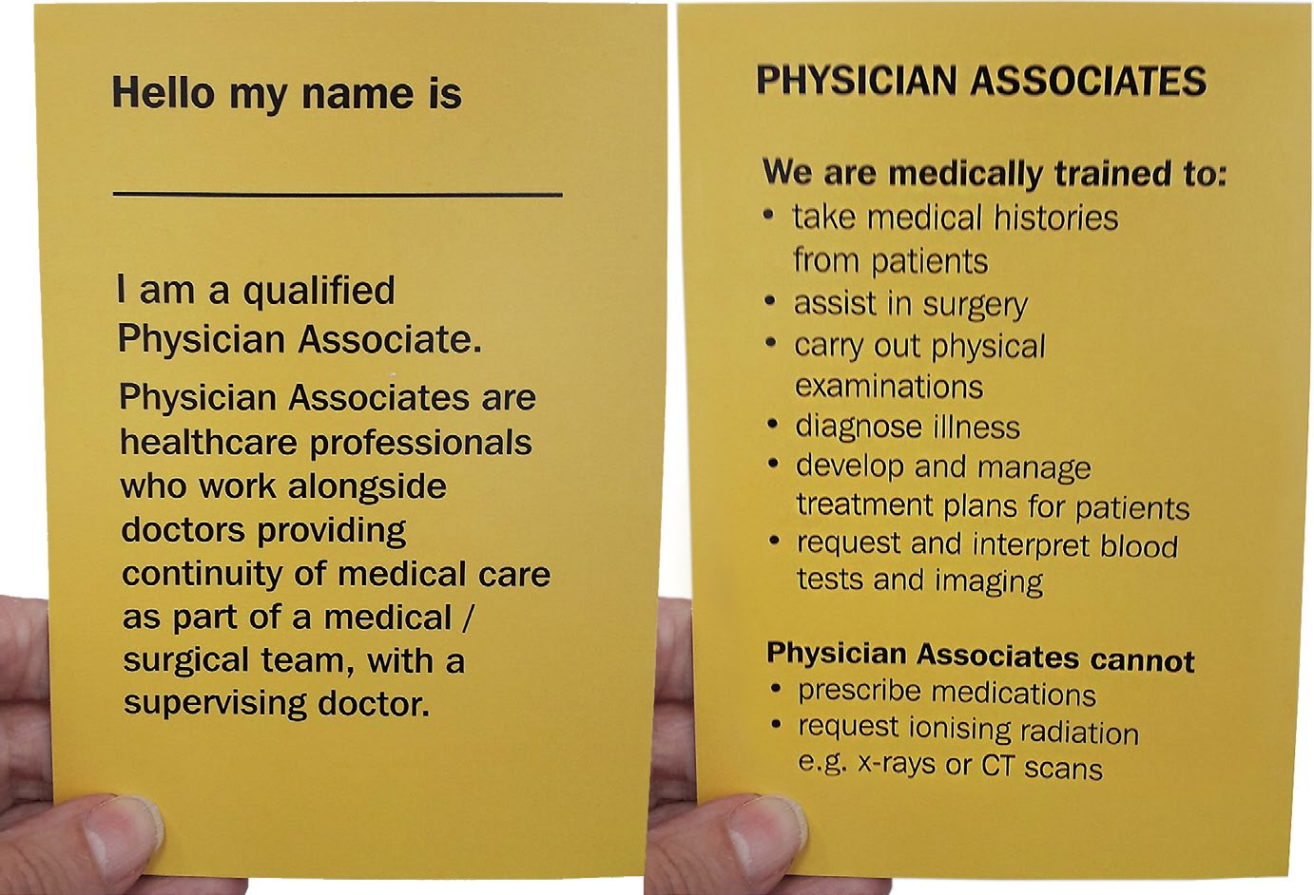
Two sides of the patient information leaflet

The intervention designed by participants in the second workshop was a two‐sided, hand‐size, card information leaflet with black wording on a yellow background (Figure 2), to be given to patients by a PA using a personal verbal explanation. The leaflet included information on the role of PAs within the medical/surgical team and what they are trained to do and cannot do. To make the intervention more patient‐centred, workshop participants designed space at the top of the leaflet where each PA could handwrite their name. Feedback in response to an intervention prototype circulated to participants after the workshop showed the prototype to be in concordance with their proposals.

### 3.2 Phase two: Feasibility testing

Sixteen patients and four patient representatives participated in interviews (Table 1). The patients interviewed were aged between 24 and 85 years. English was a second language for five participants. Three PAs were interviewed. The themes and sub‐themes extracted from analysis of the interview data sets are summarized in Table 2 and described in turn.

**Table 2:** Summary of overarching themes and sub‐themes

Overarching themes	Sub‐themes
1. Flexibility of use	‐ Leaflet read when personally appropriate
	‐ PAs adapted how they used intervention
2. Size and format liked	
3. Intervention appreciated and understood	‐ Acceptability of intervention
	‐ Understanding of the role
4. Communication of permission to engage	

#### 3.2.1 Flexibility of use

##### Leaflet read when personally appropriate

Few patient participants reported reading the leaflet when it was given to them. Most participants described delayed use; putting the leaflet aside and reading it at a later, personally appropriate time. Some mentioned looking at the leaflet when feeling less medicated and more alert, or less in pain, others when there were fewer staff encounters and they could give it attention. One female participant reported being handed the leaflet before surgery and not reading the information until afterwards when less anxious and preoccupied. Another female participant, whose first language was not English, described keeping the leaflet until her sister visited, and together they could read and discuss the content.

Although only one participant expressed criticism of when they received the leaflet, several participants suggested more suitable timing. There were marked differences, however, in their recommendations; some participants proposed the leaflet be provided at hospital admission, while others suggested later stages along the inpatient pathway.

Many participants recalled the PA introducing themselves before being handed the leaflet. For several participants, the personal introduction seemed to have facilitated their use of the leaflet.

S/he explained it a little which is probably why I kept it to read later on. (Participant 18, female, surgical)

##### PAs adapted how they used intervention

Each PA participant had their own way of using the intervention, adjusting how and when they introduced themselves and the leaflet to suit their personal style and work context. One participant preferred to complete routine patient interactions such as taking bloods and return later with the leaflet. Another participant reported introducing themselves at the start of the encounter, but not handing over the leaflet until the end alongside verbally outlining key aspects of their role. For one participant, introduction of the leaflet was initially thought discordant with the mood and tempo they liked to maintain in patient encounters, particularly since the information was unrequested. They described trying several different approaches before finding one they felt comfortable using.

Participants also reported employing a patient‐typology targeted approach. For example, one participant said they preferred not to use the intervention with patients who appeared very anxious or who had just been given bad news.

Some initial apprehension about using the intervention was expressed by all participants, particularly in relation to how patients would respond. However, use of the intervention was reported to become easier with practice and on not experiencing rejection.

It, kind of, becomes easier to give out. Whereas at the beginning you're a bit anxious like will they want it, will they avoid taking it, will they think why am I giving it to them? (PA 2)

#### 3.2.2 Size and format liked

Plaudits were expressed by many patient participants for the leaflet's handy size and card format, making it feasible to retain for reference while an inpatient, and potentially slip into wallet or handbag when leaving hospital. Favourable comments about the concise information in easy‐to‐read, legible and straightforward language also permeated responses. Some participants spoke about the accessibility of the information through use of bullet points, others talked about being able to read the leaflet without glasses.

I think the size and the amount of information on it is perfect for an introduction. (Participant 16, male, orthopaedic)

Diverse opinions were expressed about the leaflet's bright yellow colour, although it was consistently viewed as eye‐catching and attention‐grabbing. The most critical comments on the leaflet design were made with reference to its credibility and trustworthiness, since it lacked a hospital name or logo.

PA participants reported mostly favourable opinions on the size and format, although one participant felt a smaller‐size leaflet would be more portable, particularly when wearing scrubs. While the leaflet's colour was generally liked for being conspicuous, one participant reported confusion because it was the same colour as patients’ warfarin anticoagulant booklets.

#### 3.2.3 Intervention appreciated and understood

##### Acceptability of intervention

While frequently described as novel and unexpected, the intervention prompted generally favourable reactions from patient participants. They recounted feeling better informed about who was providing their care which was thought beneficial and reassuring. For example, a male participant talked about the assurance that came from being given information about the PA, after having had a succession of people treating him without knowing who they were or what they did. A female participant reported feeling safer in a strange environment as a consequence of having the PA role explained. Several participants included not only the intervention, but the hospital in their appreciation.

It tells you behind the scenes that the hospital cares about patients…is taking an interest to make sure all the patients know. (Participant 1, male, acute stroke)

Specific attention to promotion of the PA role was queried by a few participants; information on all medical team roles was considered more helpful. Nevertheless, all but two of the participants said they thought use of the intervention should continue. Two participants thought it immaterial who looked after them as an inpatient, provided they were given appropriate care.

The dominant communication of the intervention was reported to be that PAs are qualified to do a range of medical tasks within the medical team. The role and place of PAs within the team also seemed to be clearly conveyed; participants reported knowing what to expect of their PA. Although the leaflet did not explicitly state that PAs were not doctors, this was the general understanding. Some participants talked about PAs being the coordinator between patient and doctor, providing better continuity of patient care. Others suggested that the PA role had been introduced to support doctors.

To me it reads that they're trying to help the doctors on the medical side as much as possible. (Participant 4, female, orthopaedic)

While some participants reported surprise at learning PAs were not doctors, the intervention overall inspired trust and confidence in the knowledge and skills of PAs.

Most participants expressed satisfaction with the level of information provided in the leaflet. However, some participants reported wanting to ask their PA more details about the role such as the length of training required and why PAs could not prescribe medicines.

#### 3.2.4 Communication of permission to engage

The intervention generated anticipation of the informational benefits of the PA for many participants, in particular as an information source about their care and treatment. However, while the hand‐written name in the leaflet was generally perceived as a personal link to the PA, more explicit communication as to whether and how the PA could be accessed was widely advocated. Some participants spoke about making it clearer that the PA welcomed questions, others mentioned wanting permission to engage, for example by providing contact details for the PA.

It does need that little bit more information, when do you contact them, how do you contact them? (Participant 12, male, orthopaedic)

All PA participants reported being disappointed that follow‐up questions from patients in response to the intervention were fewer than anticipated. They suggested patients be facilitated to seek additional information if wanted.

Something at the bottom of the leaflet that says, “for more information please ask me”. (PA 3)

## 4 DISCUSSION

This qualitative study identified that the patient preferred method of introducing PAs was a small information leaflet provided by the PA with a personal verbal explanation. Our study demonstrated that the intervention was feasible to use in the acute hospital setting, helpful to patients in understanding the PA role and acceptable to both patients and PAs. It is the first study to develop with PPIE an intervention to introduce inpatients to a novel health professional role.

Our findings identified two key attributes of the intervention that seemed to influence feasibility and acceptability. First, its adaptability. Patient participants appeared willing and content to adjust the timing of when they read the information leaflet to suit their emotional and/or physical status. Adaptability of the intervention to fit individual style was also practised by PA participants. This positive attribute accords with existing evidence for how best to use PILs; that when used at an appropriate time and well written they can improve patient knowledge whatever the acute clinical condition.^40^ Second, the clarity and readability of the leaflet. It was credited by patient participants for clearly communicating the PA role within the medical team and what PAs are medically trained to do and cannot do. Evidence suggests that the informative value of patient leaflets is likely to be more effective if they are developed to meet health literacy needs across the population.^44^

The intervention was considered a novel and surprising approach to information provision in the hospital context by many patient participants. Although the PA participants described different ways that they introduced themselves and the information leaflet to patients, the variant used did not appear to influence the response of patient participants. It was the personal introduction of the leaflet by PAs that was generally welcomed and seemed important in encouraging patient use of the leaflet. This is in line with evidence‐based recommendations for introducing information leaflets to patients; hand‐delivered and personalized by the clinician.^40^ Leaflets on their own have been found to have limited effect, but combined oral and written information can enhance patient engagement.^45^

Our findings revealed appreciation of the intervention not only for the information provided, but because it generated reassurance of care and support. The anticipated benefits of accessing information from the PA about their personal care and treatment were attractive to several participants. These responses are congruent with findings from the literature indicating that patients receive insufficient personal information from their doctors during hospital ward rounds.^46^ The psychological benefits of patient information in the hospital context are also recognized in terms of helping patients know what to expect, thereby reducing uncertainty and anxiety.^20^, ^21^

While patient participants generally welcomed the intervention, PA participants described initial apprehension about introducing it into routine patient encounters. They anticipated some rejection of the offer of information. Clinicians’ underestimation of the amount of information that patients need and want has been recognized in the literature.^47^, ^48^

Our study also identified that it would be beneficial to clarify in the leaflet how the PA might be contacted, allowing patients to access additional information if wanted. Patient participants felt this would make the intervention more patient‐centred and enable communication of tailored and personalized treatment information. Existing evidence shows that patient leaflets providing easy to understand proactive information can invite and encourage patient participation in their care.^49^ Our study finding also reflects the patient empowerment discourse identified by Dixon‐Woods,^50^ where patients use information as a resource aiding illness management. After consideration of these findings, the Patient Research Expert Group, the lead PA and the study researchers agreed that the patient leaflet should be revised to include the phrase, ‘For more information, please ask me.’

### 4.1 Strengths and limitations

A strength of the study was the use of an experience‐based co‐design approach^34^ which supported development of an intervention tailored to patients’ experiential preferences. The use of visual and written ideas for introducing PAs has shown some promise as a methodology to elicit and explore patient preferences prior to intervention design. Facilitation of the workshops enabled a patient‐centred focus. Workshop participants themselves reported the co‐design process as unpredictable and characterized by creative disagreement, but nonetheless rewarding. Incorporation in data analysis of guidance from the literature on evaluating PILs^40^ provides evidence‐based understanding of feasibility of the designed intervention.

There are limitations to the study. Workshop participants were not involved in all decision‐making meetings, identified as a problem in other intervention development studies incorporating PPIE.^51^ Some meetings between the PA participants and a study researcher took place outside the workshops due to clinician time‐constraints, challenging the intended more equitable power‐sharing.

While we endeavoured to employ purposive qualitative patient samples to provide diversity, the use of PAs as gatekeepers may have resulted in selection bias and recruitment of convenience samples. Our study was also based on small numbers of participants recruited from one hospital study site. Nevertheless, while the findings cannot be easily generalized, they do offer insights for potential further testing.

## 5 CONCLUSION

Patient confusion with the titles and roles of new health professionals being introduced into hospital medical/surgical teams is widely recognized, alongside a positive association between patients’ ability to identify practitioners involved in their care and patient satisfaction. This paper contributes to the literature as the first study to develop a patient experience‐based intervention for introducing one of these novel roles, PAs, to hospital patients. The information leaflet was found acceptable and endorsed by patients and PAs. It was credited for clearly communicating the PA role in the medical/surgical team and that PAs are not doctors. Patients appreciated the intervention, and it seemed to have the potential to improve their hospital care experience, generating reassurance of care and support. It also raised expectations of being able to seek additional information and engage more in their care if desired. Feasibility was encouraging, supporting future larger‐scale research to evaluate the impact of the intervention on awareness and understanding of the PA role and similar interventions for other new health professional roles.

## CONFLICT OF INTEREST

The authors declare no conflicts of interest.

## AUTHOR CONTRIBUTIONS

VMD: Conceived and led the research, involved in the study design, analysis and interpretation of data and drafting of the manuscript. FT: Conceived the research, involved in the study design, undertook interviews, involved in analysis and interpretation of data and drafting of the manuscript. JO, RC, ZL: Contributed to the study design and revised the manuscript critically. SB: Involved in the study design, interpretation of data and revised the manuscript critically.

## ETHICAL APPROVAL

The study was approved by a UK NHS Research Ethics Committee (18/YH/0304). Written informed consent was obtained from each interview participant.

## Data Availability

De‐identified interview data sets analysed in the current study are available from the corresponding author on reasonable request.
